# Activation of the unfolded protein response and granulovacuolar degeneration are not common features of human prion pathology

**DOI:** 10.1186/s40478-016-0383-7

**Published:** 2016-10-28

**Authors:** Vera I. Wiersma, Wim van Hecke, Wiep Scheper, Martijn A. J. van Osch, Will J. M. Hermsen, Annemieke J. M. Rozemuller, Jeroen J. M. Hoozemans

**Affiliations:** 1 Department of Pathology, Amsterdam Neuroscience, VU University Medical Center, P.O. Box 7057 , 1007 MB Amsterdam, The Netherlands; 2 Dutch Surveillance Centre for Prion Diseases, University Medical Center Utrecht, Utrecht, The Netherlands; 3 Department of Clinical Genetics and Alzheimer Center, VU University Medical Center, Amsterdam, The Netherlands; 4 Departments of Functional Genomics, Center for Neurogenomics and Cognitive Research, VU University, Amsterdam, The Netherlands

**Keywords:** dsRNA-activated protein kinase-like ER kinase, Granulovacuolar degeneration, Inositol-requiring enzyme 1α, Prion disease, Tau, Unfolded protein response

## Abstract

Human prion diseases are fatal neurodegenerative disorders with a genetic, sporadic or infectiously acquired aetiology. Neuropathologically, human prion diseases are characterized by deposition of misfolded prion protein and neuronal loss. In post-mortem brain tissue from patients with other neurodegenerative diseases characterized by protein misfolding, including Alzheimer’s disease (AD) and frontotemporal lobar degeneration with tau pathology (FTLD-tau), increased activation of the unfolded protein response (UPR) has been observed. The UPR is a cellular stress response that copes with the presence of misfolded proteins. Recent studies have indicated that UPR activation is also involved in experimental models of prion disease and have suggested intervention in the UPR as a therapeutic strategy. On the other hand, it was previously shown that the active form of the UPR stress sensor dsRNA-activated protein kinase-like ER kinase (PERK) is not increased in post-mortem brain tissue samples from human prion disease cases. In the present study, we assessed the active form of another UPR stress sensor, inositol-requiring enzyme 1α (IRE1α), in human post-mortem frontal cortex of a large cohort of sporadic, inherited and acquired prion disease patients (*n* = 47) and non-neurological controls. Immunoreactivity for phosphorylated IRE1α was not increased in prion disease cases compared with non-neurological controls. In addition, immunoreactivity for phosphorylated PERK was unaltered in human prion disease cases included in the current cohort. Moreover, no difference in the extent of granulovacuolar degeneration, a pathological feature associated with the presence of UPR activation markers, was detected. Our data indicate that, in contrast to AD and primary tauopathies, activation of the UPR is not a common feature of human prion pathology.

**Electronic supplementary material:**

The online version of this article (doi:10.1186/s40478-016-0383-7) contains supplementary material, which is available to authorized users.

## Introduction

Human prion diseases are rare, rapidly progressive, invariably lethal neurodegenerative diseases, symptomatically characterized by severe memory impairment and a general decline in cognitive functions, which may include motor, linguistic, executive and social skills [1]. Most often, human prion diseases have a sporadic aetiology (e.g. sporadic Creutzfeldt-Jakob disease (sCJD)), but hereditary (e.g. fatal familial insomnia (FFI) and Gerstmann-Sträussler-Scheinker syndrome (GSS)), and infectiously acquired (e.g. iatrogenic CJD (iCJD), kuru and variant CJD (vCJD)) forms of the disease also exist [2]. The clinical duration varies between the subgroups of the disease, with a mean disease duration ranging from 2 to 3 months for sCJD, 12 months for kuru and 5 years for GSS [3]. Prion diseases have also extensively been described in animals, including bovine spongiform encephalopathy (BSE) in cattle and scrapie in sheep [2]. At the neuropathological level human prion diseases are characterized by the accumulation of pathological prion protein (PrP^Sc^), neuronal loss, astrogliosis and spongiosis [4, 5]. PrP^Sc^ arises after the post-translational conformational conversion of the cellular prion protein (PrP^C^). PrP^C^ is a constitutively expressed protein of uncertain function that contains a glycosylphosphatidylinositol (GPI) anchor, facilitating its association with the cell membrane. During human prion diseases PrP^C^ is converted into insoluble, β-sheet rich PrP^Sc^ aggregates that are usually resistant to digestion by proteinase-K. This pathological PrP^Sc^ conformer exhibits intriguing characteristics that, once formed, ensure conversion of native PrP^C^ into PrP^Sc^ and propagation of pathology to neighbouring cells [6–10].

Previous research has pointed out that the endoplasmic reticulum (ER), an organelle essential for protein processing, might constitute a link between prion pathology and neurodegeneration [11–18]. If the functioning of the ER is disturbed, for example when un- or misfolded proteins accumulate in the lumen of the ER or in case of an aberrant calcium concentration, an adaptive programme called the unfolded protein response (UPR) is induced [19]. The UPR aims to protect the cell by restoring protein homeostasis. To this end, three canonical signalling cascades are activated by detachment of the ER luminal chaperone BiP/Grp78 from the ER stress sensors dsRNA-activated protein kinase-like ER kinase (PERK), activating transcription factor 6 (ATF6) and inositol-requiring enzyme 1 α (IRE1α). The net effect of UPR activation is relief from the ER stress, via attenuation of translation and upregulation of genes that promote chaperone synthesis and protein degradation. However, prolonged activation of the UPR can drive its outcome from adaptation to apoptosis, as programmed cell death can then be induced via among others the pro-apoptotic proteins CCAAT/enhancer-binding-protein homologous protein (CHOP), jun NH_2_-terminal kinase (JNK) and caspase-12 [19–22]. In human brain tissue activation of the UPR has been detected in several neurodegenerative diseases including Alzheimer’s disease (AD), frontotemporal lobar degeneration with tau pathology (FTLD-tau), Parkinson’s disease (PD) and amyotrophic lateral sclerosis (ALS) (for review see [23]). Prominent UPR activation is observed in AD and FTLD-tau in close association with the accumulation of phosphorylated tau [24–26]. In addition, different UPR activation markers are associated with granulovacuolar degeneration (GVD). GVD is characterized by basophilic granules surrounded by a clear zone measuring 1–5 μm in diameter, occurring predominantly in hippocampal neurons in AD and marked by the increased appearance of a variety of proteins related to cell stress [25, 27].

Studies investigating UPR activation in human prion diseases are very limited and have so far yielded inconclusive results. One study reported increased expression of BiP and several other ER chaperones in post-mortem brain samples of sCJD and vCJD patients [12]. On the other hand, immunohistochemistry on post-mortem brain tissue of human prion disease patients could not detect activation of the PERK pathway [28]. In order to investigate the possibility that other routes of the UPR are activated in human prion disease, we assessed the presence of phosphorylated IRE1α (pIRE1α) on post-mortem frontal cortex tissue of an extensive cohort of sporadic, inherited and acquired human prion disease patients. To align with the earlier report, immunohistochemistry for phosphorylated PERK (pPERK) was performed in our independent cohort. In addition, we studied the presence of a marker for GVD, as a pathological hallmark associated with the increased presence of UPR activation and cell stress-related markers [27].

## Materials and methods

### Post-mortem brain tissue

Post-mortem brain tissue of human prion disease patients, AD patients and age matched non-demented controls was obtained from the Dutch Surveillance Centre for Prion Diseases (Utrecht, The Netherlands), the Department of Pathology VU University Medical Centre (Amsterdam, The Netherlands) and the Netherlands Brain Bank (Amsterdam, The Netherlands). Autopsies were performed after informed consent and were approved by the local ethics committees. Neuropathological diagnosis was performed and confirmed according to standard procedures as described previously [29]. All prion disease cases included in this study showed presence of PrP^Sc^ deposits after immunohistochemical staining using the 3F4 antibody. Genetic sequencing for mutations in *PRNP* and the codon 129 subtype (methionine/valine polymorphism) and Western blotting for PrP^Sc^ typing (type 1 or 2, depending on the size of the proteinase-K resistant part of PrP^Sc^) were performed at the Dipartimento di Scienze Neurologiche, Università di Bologna (Bologna, Italy) [30, 31]. Classification of sCJD subtypes was conducted according to Parchi et al. [32]. Information on all cases used in the present study is listed in Table 1. In summary, 5 GSS patients (mean age 51 years), 3 FFI patients (mean age 52 years), 3 vCJD patients (mean age 30 years), 3 iCJD patients (mean age 59 years), 1 patient with prion protein cerebral amyloid angiopathy (PrP-CAA) (57 years), 1 patient with variably protease-sensitive prionopathy (VPSPr) (47 years) and 31 sCJD patients (mean age 66 years) comprising different sCJD subtypes, including 2 panencephalopathic sCJD patients, were included in the present study. Non-neurological control cases (*n* = 9, mean age 61 years) and sporadic or familial AD patients (sporadic AD: *n* = 4, mean age 67 years, familial AD: *n* = 1, 29 years) were used as negative and positive controls for the presence of UPR activation markers, respectively. In this study, there were no differences in the processing of post-mortem brain tissue derived from human prion disease patients, AD patients and control cases.

**Table 1:** Cases included in the present study

Case	Age (years)	Gender	Neuropathological diagnosis	Mutation in *PRNP* or other gene	Codon 129 genotype	PrP type	Braak stage NFT^a^	Disease duration (months)	PMI (hours)	Cause of death
1	51	F	Ctrl	-	-	-	-	-	<24	Traffic accident
2	66	F	Ctrl	-	-	-	-	-	<48	Haemorrhagic shock
3	82	F	Ctrl	-	-	-	-	-	NA	Myocardial infarct
4	52	M	Ctrl	-	-	-	-	-	<24	Suicide
5	70	M	Ctrl	-	-	-	-	-	<48	Asphyxia
6	60	F	Ctrl	-	-	-	-	-	7.30	Infection
7	60	F	Ctrl	-	-	-	-	-	6.50	Metastasized mamma carcinoma
8	55	M	Ctrl	-	-	-	-	-	7.30	Euthanasia with oesophageal carcinoma
9	57	F	Ctrl	-	-	-	-	-	7.40	Metastasized bladder carcinoma
10	59	M	GSS	7-OPRI [59]	MV	1	NA	7	NA	Cerebral pathology
11	57	M	GSS	7-OPRI [59]	VV	1	I	65	<48	Cerebral pathology
12	42	F	GSS	5-OPRI [60]	MM	1/2	0	92	<24	Cerebral pathology
13	52	M	GSS	G131V [61]	MV	Not 1 or 2	III	192	5.45	Cerebral pathology
14	45	F	GSS	Q227X [39]	MV	Not 1 or 2	VI	72	<6	Cerebral pathology
15	57	F	PrP-CAA	Y226X [39] and D178N	MV	NA	0	27	NA	Cerebral pathology
16	36	F	FFI	D178M	MM	2	NA	48	<24	Cerebral pathology
17	61	M	FFI	D178N	MM	2	III	7	<48	Cerebral pathology
18	58	M	FFI [62]	D178N	MM	2	NA	6	<24	Cerebral pathology
19	16	M	vCJD	-	MM	2	0	9	<144	Cerebral pathology
20	26	F	vCJD [63]	-	MM	2	0	20	<24	Cerebral pathology
21	49	F	vCJD	-	MM	2	NA	15	<48	Cerebral pathology
22	54	M	iCJD	-	MM	1	0	4	<72	Cerebral pathology
23	66	M	iCJD	-	MV	1	NA	9	NA	Cerebral pathology
24	58	M	iCJD	-	MV	1	I	4	<24	Cerebral pathology
25	55	F	sCJD	-	MV	2	0-I	16	<120	Cerebral pathology
26	75	F	sCJD	-	MM	1/2	III-IV	12	NA	Cerebral pathology
27	64	F	sCJD	-	MV	2	0	8	<144	Cerebral pathology
28	61	F	sCJD	-	MV	2	0	20	NA	Cerebral pathology
29	68	F	sCJD	-	MV	2	0	26	<216	Cerebral pathology
30	59	F	sCJD	-	MV	2	0	22	<216	Cerebral pathology
31	52	F	sCJD	-	VV	1	I-II	3	<72	Cerebral pathology
32	60	M	sCJD	-	VV	2	0	6	<48	Cerebral pathology
33	79	F	sCJD	-	MV	2	III	12	<24	Cerebral pathology
34	50	F	sCJD	-	MM/MV	1/2	0	4	<48	Cerebral pathology
35	68	M	sCJD	-	MM/MV	1	0	1	<24	Cerebral pathology
36	81	M	sCJD	-	MM/MV	1	I	2	<24	Cerebral pathology
37	62	F	sCJD	-	MM/MV	1/2	I-II	1	<24	Cerebral pathology
38	62	F	sCJD	-	VV	2	I-II	4	<20	Cerebral pathology
39	60	F	sCJD	-	MV	2	I	20	<5	Cerebral pathology
40	62	M	sCJD	-	MV	2	I-II	16	<24	Cerebral pathology
41	77	F	sCJD	-	VV	2	II	5	<48	Cerebral pathology
42	81	M	sCJD	-	MM	2	I-II	36	<24	Cerebral pathology
43	73	F	sCJD	-	MV	2	I	10	<120	Cerebral pathology
44	70	F	sCJD	-	VV	2	I-II	6	<24	Cerebral pathology
45	73	F	sCJD	-	MM/MV	1/2	0	2	<24	Cerebral pathology
46	57	F	sCJD	-	MM/MV	NA	I	24	<192	Cerebral pathology
47	52	F	sCJD	-	MV	2	0	7	<24	Cerebral pathology
48	67	F	sCJD	-	MM	1/2	0	2	<24	Cerebral pathology
49	82	F	sCJD	-	MM	1	III	2	<96	Cerebral pathology
50	62	M	sCJD	-	VV	2	0	5	<24	Cerebral pathology
51	83	F	sCJD	-	MM	1	II	2	<24	Cerebral pathology
52	59	F	sCJD	-	MV	1/2	I	36	<24	Cerebral pathology
53	76	M	sCJD	-	MV	2	I	4	<24	Cerebral pathology
54	63	F	sCJD (p.enceph.) [64]	-	MV	2	0	36	NA	Cerebral pathology
55	64	F	sCJD (p.enceph.) [64]	-	MM	1	0	12	<96	Cerebral pathology
56	47	M	VPSPr [65]	-	VV	Not 1 or 2	I	20	<120	Cerebral pathology
57	65	F	Sporadic AD	-	-	-	VI	48	<24	Cerebral pathology
58	69	M	Sporadic AD	-	-	-	V-VI	48	<48	Cerebral pathology
59	29	F	Familial AD	S170F in *PSEN1*	-	-	VI	72	<48	Cerebral pathology
60	83	M	Sporadic AD (Hip)	-	-	-	III	120	<12	Cerebral pathology
61	84	F	Sporadic AD (Hip)	-	-	-	VI	84	<12	Cerebral pathology

For this study the frontal lobe was used unless indicated otherwise

*Abbreviations*: *M* Male, *F* Female, *Ctrl* Control, *GSS* Gerstmann–Sträussler–Scheinker syndrome, *PrP-CAA* PrP-Cerebral amyloid angiopathy, *FFI* Fatal Familial Insomnia, *vCJD* Variant CJD, *iCJD* Iatrogenic CJD, *sCJD* Sporadic CJD, *sCJD (p.enceph.)* Sporadic CJD panencephalopathic subtype, *VPSPr* variably protease-sensitive prionopathy, *AD* Alzheimer’s disease, *OPRI* Octapeptide repeat insertion, *M* Methionine, *V* Valine, *PMI* Post-mortem interval, *Hip* Hippocampal sections used instead of frontal sections, *NA* Not available

^a^Braak stage for NFT was used to describe the severity of tau pathology. However, since in prion diseases tau pathology can also be secondary to PrP^Sc^ amyloidosis instead of Aβ amyloidosis, this staging does not represent real Braak and Braak classification, but rather an indication of the severity of tau pathology, described as if it were an AD patient. Additionally, tau and Aβ pathology in the frontal cortex were assessed by our own immunohistochemical stainings

### Immunohistochemistry

Formalin-fixed, paraffin embedded frontal cortex (F2) or hippocampal sections of 5 μm were cut and mounted on microscope slides (Leica Xtra adhesive slides, Leica Microsystems, Rijswijk, The Netherlands or SuperFrost Plus microscope slides, VWR, Leuven, Belgium). After deparaffinization and rehydration, endogenous peroxidase activity was blocked in 0.3 % H_2_O_2_ in methanol for 30 min. An antigen retrieval step of 10 min pre-treatment with heated sodium-citrate buffer (10 mM/L, pH 6.0) was performed for the primary antibodies against pIRE1α, casein kinase 1 delta (CK1δ), phosphorylated pathological tau (AT8) and β-amyloid peptide (Aβ, IC16). For detection of PrP^Sc^ using the 3F4 antibody, sections were pre-treated for 5 min with formic acid followed by quenching of endogenous peroxidase activity and pre-treatment in heated citric acid (10 mM/L, pH 6.0) for 10 min. No antigen retrieval procedure was carried out for detection of pPERK. All primary antibodies were diluted in DAKO antibody diluent (DAKO, Glostrup, Denmark) (Table 2). Negative controls were obtained by omission of primary antibody from a case established to show immunoreactivity with the omitted antibody. Primary antibody incubation was performed overnight at 4 °C. As secondary step, sections were incubated with the EnVision detection system (goat anti-mouse/rabbit horseradish peroxidase (HRP), DAKO) for 30 min at room temperature. Between incubation steps, sections were rinsed with phosphate buffered saline (PBS). Sections were incubated for 5 min with the chromogen 3,3′-diaminobenzidine (DAB, EnVision Detection system/HRP, DAKO) to visualize immunoreactivity. Nuclei were counterstained with haematoxylin. Hereafter, slides were dehydrated and mounted using the non-aqueous mounting medium Quick-D (Klinipath, Duiven, The Netherlands).

**Table 2:** Overview of the primary antibodies used in the present study to visualize UPR activation, GVD and pathological proteins

Antibody	Species	Dilution	Antigen	Source
pPERK	Rabbit	1:800	PERK phosphorylated at Thr981	Santa Cruz Biotechnology
pIRE1α	Rabbit	1:10.000	IRE1α phosphorylated at Ser724	Novus Biologicals
CK1δ	Mouse	1:25	Amino acids 296–355 of CK1δ	Santa Cruz Biotechnology
AT8	Mouse	1:800	Tau phosphorylated at Ser202 and Thr205	Pierce Biotechnology
IC16	Mouse	1:800	N-terminal amino acids 1–6 of Aβ	Kind gift of Prof. Dr. Korth, Heinrich Heine University, Düsseldorf, Germany [36]
3F4	Mouse	1:800	Amino acids 109–112 of protease sensitive and protease insensitive PrP	Covance

Primary antibodies used in the present study. The name of the primary antibody, the species of the host animal the primary antibody was raised in, the antigen recognized by the primary antibody and the source are listed

### Analysis of immunohistochemical stainings and statistics

Immunoreactivity for pIRE1α, pPERK and CK1δ was quantified by counting the amount of positive neurons in the total grey matter of each section using either a 10× or 25× objective (12.5× ocular) of a Zeiss microscope (Zeiss, Oberkochen, Germany). The surface area (in cm^2^) of the grey matter of each section was assessed using QProdit software (Leica Microsystems) and used to correct pIRE1α, pPERK and CK1δ scores. Using the 2.5×, 10× and 25× objective (12.5× ocular) of a Zeiss microscope, immunoreactivity for phosphorylated tau, Aβ and prion protein was descriptively analysed with reference to the literature [33–36]. Semi-quantitative scores of the tau and Aβ burden were assigned to each case (Table 3 and Additional file 1: Figure S1).

**Table 3:** Overview of the results obtained from immunohistochemical stainings for pIRE1α, pPERK, CK1δ, Aβ and phosphorylated tau

Case	Neuropathological diagnosis	pIRE1α	pPERK	CK1δ	Aβ deposits	pTau (small inclusions/neuritic changes)	pTau (tangle-like)
1	Ctrl	0	0	0	-	-	-
2	Ctrl	0	0	0	-	-	-
3	Ctrl	0	0	0	-	−/+	-
4	Ctrl	0	0	0	-	-	-
5	Ctrl	0	0	0	-	-	-
6	Ctrl	0	0	0	++	−/+	-
7	Ctrl	0	0	0	-	-	-
8	Ctrl	0	0	0	-	−/+	-
9	Ctrl	0	0	0	+	-	-
10	GSS	0	0	2	+	+++	-
11	GSS	0	0	3	++	++/+++	-
12	GSS	0	0	0	-	−/+	-
13	GSS	0	0	0	-	-	-
14	GSS	0	10	21	-	+++	+++
15	PrP-CAA	1	0	0	-	++	-
16	FFI	0	0	2	-	−/+	-
17	FFI	2	0	1	++	++/+++	-
18	FFI	0	0	2	-	−/+	-
19	vCJD	0	0	0	-	+++	-
20	vCJD	1	0	4	-	+++	-
21	vCJD	0	0	2	-	+++	-
22	iCJD	0	0	0	-	−/+	-
23	iCJD	0	0	0	-	++	-
24	iCJD	0	0	1	+	++	-
25	sCJD	0	0	0	-	+	-
26	sCJD	0	1	0	+++	++/+++	++
27	sCJD	0	0	0	-	++	-
28	sCJD	0	0	0	+	++	-
29	sCJD	0	0	0	-	++/+++	-
30	sCJD	0	0	1	-	++	-
31	sCJD	0	0	1	-	++	-
32	sCJD	1	0	0	-	+	-
33	sCJD	0	0	1	+++	+	+
34	sCJD	0	0	2	-	+	-
35	sCJD	0	0	2	-	+	-
36	sCJD	0	0	6	++	++/+++	++/+++
37	sCJD	0	0	2	++	++	-
38	sCJD	1	0	1	++	++	-
39	sCJD	0	0	0	+	++	-
40	sCJD	1	0	0	+	+	-
41	sCJD	0	0	2	-	−/+	-
42	sCJD	1	0	0	++	++	-
43	sCJD	0	0	0	-	−/+	-
44	sCJD	4	0	2	+++	++	-
45	sCJD	0	0	0	-	−/+	-
46	sCJD	0	0	1	-	++/+++	-
47	sCJD	0	0	0	-	−/+	-
48	sCJD	0	0	0	-	−/+	-
49	sCJD	0	0	0	++	−/+	−/+
50	sCJD	0	0	0	-	+	-
51	sCJD	0	0	1	++	−/+	-
52	sCJD	0	0	1	-	−/+	-
53	sCJD	0	0	0	+	+	-
54	sCJD (p.enceph.)	0	0	0	-	++	-
55	sCJD (p.enceph.)	0	0	0	-	−/+	-
56	VPSPr	0	0	0	-	+	-
57	Sporadic AD	0	0	5	++	++	+
58	Sporadic AD	0	2	1	++	++	+
59	Familial AD	14	83	204	+++	+++	+++

pIRE1α, pPERK and CK1δ scores were obtained by quantification of the amount of positive neurons. The values are corrected for the surface area of each section. The value represents the amount of positive neurons per cm^2^. All values are rounded to the nearest whole number. Aβ and tau scores were semi-quantitatively assessed. See Additional file 1: Figure S1 for representative examples of the +, ++ and +++ scores of all three classes. Positive controls from AD hippocampus are not depicted in this table

*Abbreviations*: *pTau* phosphorylated tau, *p. enceph.* panencephalopathic subtype

## Results

### Assessment of concomitant AD pathology

**Fig. 1:**
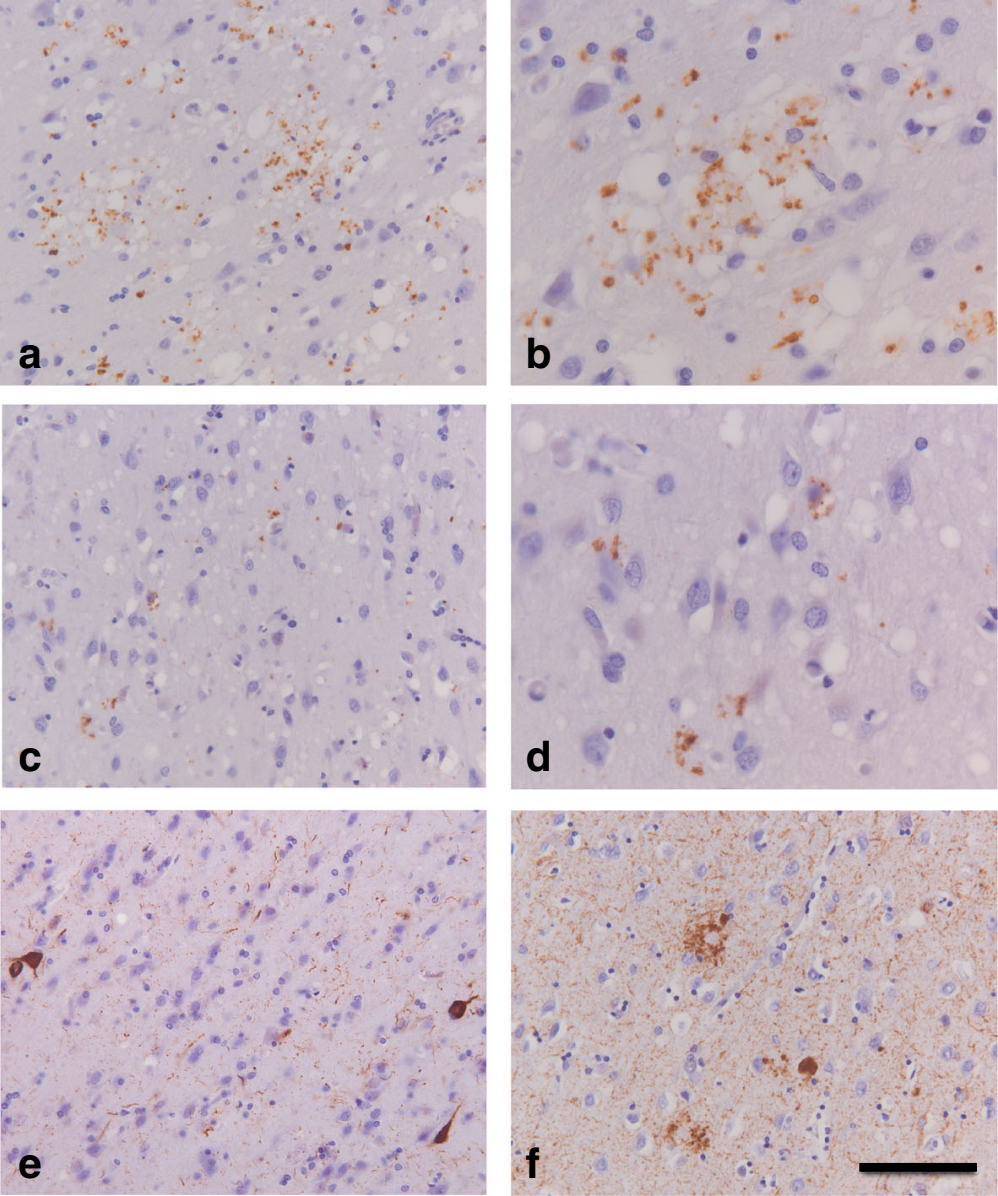
Immunohistochemical detection of phosphorylated tau in tissue of human prion disease patients. **a** Occurrence of hyperphosphorylated tau (AT8 antibody) in the frontal cortex of a vCJD case (case #20). **b** Hyperphosphorylated tau is associated with “grape-like” clusters of spongiosis in the frontal cortex of a vCJD case (case #20). **c** Occurrence of hyperphosphorylated tau in the frontal cortex of a sCJD case (case #46). **d** Hyperphosphorylated tau in the frontal cortex of a sCJD case (case #46) showing a neuronal/perineuronal localization. **e** Occurrence of hyperphosphorylated tau positive neuropil threads and tangle-like changes in a sCJD case (case #26). **f** Immunohistochemical detection of hyperphosphorylated tau in the frontal cortex of an AD case (case #59) showing staining of neuritic plaques, neuropil threads and neurofibrillary tangle-like structures. **a**-**f** Brown staining with DAB, *blue* staining of the nucleus with haematoxylin. *Bar* **a**, **c**, **e **,**f** 100 μm; **b**, **d** 20 μm

Previous reports have shown UPR activation in close association with tau pathology in cases with AD and FTLD-tau [24, 25]. In addition, it has earlier been shown that the UPR activation marker pPERK can only be detected in human prion disease cases with concomitant AD pathology [28]. In the present study we controlled for the presence of concomitant AD pathology in the frontal cortex of human prion disease cases using the Braak staging for neurofibrillary tangles, which was applied to almost all prion disease cases included in this study (Table 1). Most prion disease cases had a Braak stage between 0 and III, indicating that neurofibrillary tau tangles were absent or very mildly present in the frontal cortex. The only exception was case #14, which represented a Braak stage VI (Table 1). In addition, the presence of the pathological proteins involved in AD, Aβ and (phosphorylated) tau, was assessed by immunohistochemical staining on frontal cortex sections adjacent to the sections used for assessment of UPR activation markers. Approximately 68 % of the human prion disease patients presented with some level of Aβ deposition in the frontal cortex (Table 3). Phosphorylated tau positive lesions visualized by the AT8 antibody were separately assessed into two classes: (1) small, hyperphosphorylated tau positive neuritic and extracellular inclusions, a staining pattern previously reported to be associated with prion pathology (Fig. 1 a-d) [34, 37], and (2) tau positive neurons presenting a phosphorylated tau staining pattern akin to pretangles and neurofibrillary tangles (Fig. 1 e, f). Almost all prion disease cases showed at least some level of small phosphorylated tau positive neuritic and extracellular inclusions, while 51 % of the prion disease cases showed moderate to severe presence of these prion pathology induced changes in tau (Table 3). On the other hand, phosphorylated tau positive neurons showing (pre-)tangle immunostaining could be detected in the frontal cortex of only 11 % of prion disease cases (Table 3).

### Immunohistochemical analysis of UPR activation markers

**Fig. 2:**
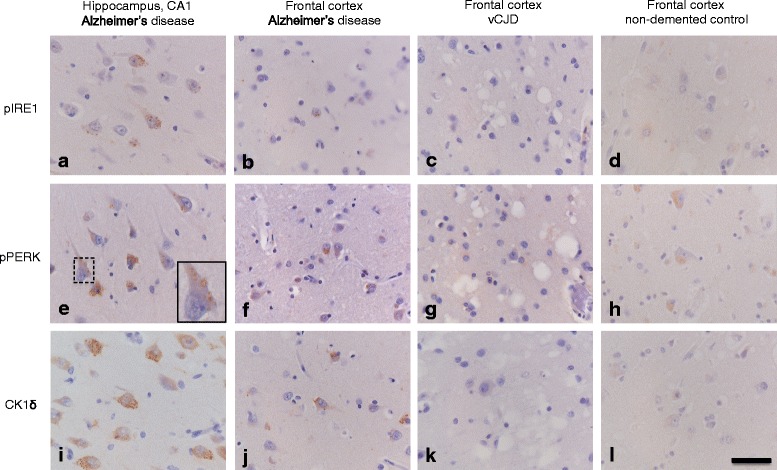
Immunohistochemical detection of pIRE1α, pPERK, and CK1δ in AD, vCJD and control brain tissue. Representative pictures are shown of the immunohistochemical detection of pIRE1α (**a**-**d**), pPERK (**e**-**h**) and CK1δ (**i**-**l**) in the hippocampus or frontal cortex of AD, vCJD and non-demented control cases. Immunohistochemical detection of pIRE1α (**a**), pPERK (**e**) and CK1δ (**i**) in neurons in the CA1 region of the hippocampus of a sporadic AD case (case #61). Immunohistochemical detection of pIRE1α (**b**), pPERK (**f**) and CK1δ (**j**) in the frontal cortex of a familial AD case (case #59). Immunohistochemical detection of pIRE1α (**c**), pPERK (**g**) and CK1δ (**k**) in the frontal cortex of a case with vCJD (case #20). Immunohistochemical detection of pIRE1α (**d**), pPERK (**h**) and CK1δ (**i**) in the frontal cortex of a non-demented control case (case #5). The inset (**e**) shows a typical granular staining which can be referred to as GVD. Immunohistochemical detection is visualized by DAB (*brown* staining) and nuclei are counterstained with haematoxylin (*blue* staining). *Bar* **a**-**l** 50 μm

Previously, we reported increased pIRE1α immunoreactivity in AD hippocampus [25]. For this study, AD hippocampus was used as a positive control. In addition, frontal cortex of sporadic and familial AD cases was included in the cohort. In AD hippocampus pIRE1α was visible in small granules, puncta or vacuoles, reminiscent of GVD, which was most prominently present in the CA1 region and subiculum of AD patients (Fig. 2a). A similar staining pattern, although to a lesser extent, was observed in the frontal cortex of a familial AD case (Fig. 2b; Table 3). No pIRE1α immunoreactive neurons were observed in the frontal cortex of two sporadic AD cases and non-neurological controls (Fig. 2d; Table 3). Immunoreactivity for pIRE1α was investigated in frontal cortex sections of a cohort of sporadic, inherited and acquired human prion diseases (Table 3). Immunoreactivity for pIRE1α could hardly be detected in the frontal cortex of human prion disease cases (Fig. 2c; Additional file 2: Figure S2; Table 3). No difference in the occurrence of pIRE1α immunoreactive neurons was observed between prion disease cases and the non-neurological control group.

In addition, pPERK immunoreactivity was assessed in order to relate our findings to the previous reported absence of activation of the PERK pathway in human prion diseases [28]. pPERK was clearly detectable in AD hippocampus and associated with GVD (Fig. 2e). Immunoreactivity for pPERK was also observed in the frontal cortex of one sporadic AD case and a familial AD case in GVD-like structures (Fig. 2f, h; Table 3). Compared with frontal cortex derived from non-neurological control cases, pPERK immunoreactivity was not increased in prion disease cases, with the exception of a single case (#14) (Fig. 2g; Additional file 2: Figure S2; Table 3).

Since pIRE1α and pPERK are associated with GVD, we assessed the presence of CK1δ, an accepted immunohistochemical marker for GVD [27, 38], in human prion disease cases. Immunohistochemical staining of the hippocampus of sporadic AD cases and frontal cortex of a familial AD case demonstrated abundant neurons containing CK1δ immunoreactive granules (Fig. 2i, j; Table 3). Few neurons containing CK1δ positive granules could be detected in the frontal cortex of sporadic AD cases (Table 3). CK1δ immunoreactivity was not detectable in non-neurological control cases (Fig. 2l). No marked increase in CK1δ immunoreactivity was observed in the frontal cortex of human prion disease cases compared to non-demented controls, except for case #14 (Fig. 2k; Additional file 2: Figure S2).

To ensure that special handling of prion infected brain tissue and a relative long post mortem delay could affect the detection of the phospho-epitopes of IRE1 and PERK, pIRE1 and pPERK immunoreactivity was assessed on AD hippocampus tissue that underwent the same handling and had similar or longer post-mortem delay as about half of all prion disease cases included in this study. The hippocampus of an AD case (case# 57) that followed the same handling as all prion disease cases showed a clearly detectable pIRE1 and pPERK immunostaining in neurons located in the subiculum (Additional file 3: Figure S3A and B). The intensity and number of pIRE1 and pPERK immunoreactive neurons were comparable with previous observations on AD subiculum from an independent cohort with relatively short post-mortem delays [25]. In additional immunostainings performed on a CJD case (case #42) with a similar post-mortem delay, pIRE1 or pPERK were undetectable in the subiculum (Additional file 3: Figure S3C and D).

### pPERK activation and GVD in an atypical GSS case with secondary neurofibrillary tau pathology

**Fig. 3:**
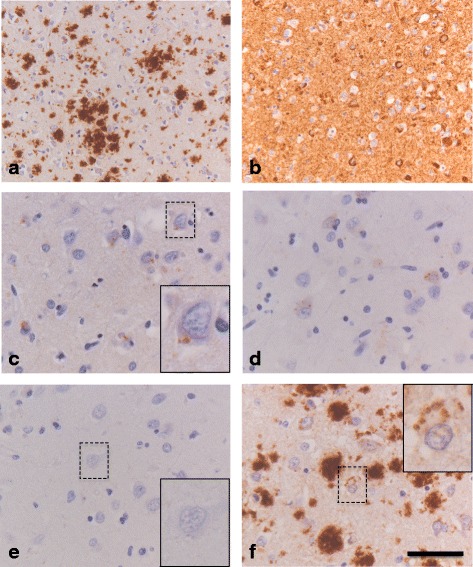
Presence of UPR activation markers and GVD in the frontal cortex of an unusual GSS case carrying a *PRNP* Q227X mutation (case #14). **a** Immunohistochemical detection of PrP using the 3F4 antibody in the frontal cortex. **b** Detection of hyperphosphorylated tau (AT8 antibody) in the frontal cortex. **c** Localization of pPERK in GVD structures (see inset) in the frontal cortex. **d** Immunohistochemical detection of CK1δ shows staining of GVD structures in the frontal cortex. **e** Immunohistochemical staining for pIRE1α shows absence of pIRE1α immunoreactivity. **f** Immunohistochemical detection of PrP using the 3F4 antibody in the frontal cortex shows intraneuronal detection in granular structures resembling GVD. Insets show higher magnification of indicated area. **a**-**f** Brown staining with DAB, blue staining of the nucleus with haematoxylin. *Bar* **a**, **b** 100 μm; **c**-**f** 20 μm

Although no significant differences were observed between non-neurological control and prion disease cases regarding the presence of UPR activation markers, one GSS case presented with relatively high numbers of neurons immunoreactive for pPERK and CK1δ (case #14). As described previously [39], this atypical GSS case died at the age 45 years, 72 months after disease onset. This patient displayed a hereditary form of human prion disease with an unusual GSS phenotype. The genetic defect could be traced back to the insertion of a thymine in the coding region of *PRNP* (Q227X), causing a premature stop codon and thus a truncated prion protein. Many PrP amyloid plaques (no Aβ deposits) and severe tau pathology were observed in the frontal cortex (Fig. 3a, b). Overall this case was staged with Braak VI for neurofibrillary tangles (NFT) (Table 3). In the frontal cortex of this patient, pPERK immunoreactivity in the form of GVD-like granules was observed (Fig. 3c). This finding was underscored by the presence of GVD bodies marked by CK1δ in adjacent cortical sections of this case (Fig. 3d). Remarkably, we were unable to detect pIRE1α immunoreactivity in this patient (Fig. 3e). Interestingly, detection of PrP^Sc^ using the 3F4 antibody also revealed intracellular immunostaining resembling a structure similar to GVD (Fig. 3f).

## Discussion

Many neurodegenerative diseases are characterized by the deposition of misfolded protein aggregates. Therefore, it is suggested that these diseases share the involvement of similar cellular signalling mechanisms associated with protein folding and clearance. Previously, we have demonstrated increased presence of several UPR activation markers, including pPERK and pIRE1α, in post-mortem brain tissue of AD, FTLD-tau and PD [24, 40, 41]. In the present study, we aimed to further elucidate the involvement of the UPR in human prion pathology. To this end, we selected cases from the Dutch cohort of human prion disease patients [29] and performed immunohistochemistry to visualize UPR activation markers on post-mortem frontal cortex sections. Immunoreactivity for pIRE1α and pPERK was not increased in human prion disease cases compared to non-neurological controls. In addition, immunoreactivity for CK1δ, a marker associated with GVD and cellular stress, was not elevated in human prion disease cases.

Earlier observations indicate a close spatiotemporal relationship between UPR activation and the presence of tau pathology [24–26]. Several mechanistic links between tau pathology and UPR activation have been described [42–49]. Previously, Unterberger and colleagues reported that immunoreactivity for pPERK in human prion diseases was observed to be associated with tau pathology only in cases with concomitant AD pathology [28]. Human prion disease patients often present modest tau positive lesions that are believed to be secondary to the prion pathology itself. This type of tau amyloidosis comprises small neuritic and extracellular inclusions consisting of hyperphosphorylated tau [34, 37]. In the present study, almost all human prion disease cases presented this type of tau pathology in the absence of profound UPR activation, as assessed by the UPR activation markers pIRE1α and pPERK. The data presented in this study suggest that this modest type of tau pathology secondary to prion pathology is not functionally linked to UPR activation in human prion diseases. In contrast, other reports on human neuropathology do support the involvement of UPR activation in neurodegenerative diseases with extensive tau pathology [23]. Interestingly, Radford and colleagues showed neuroprotective effects of a selective PERK inhibitor in a mutant tau transgenic mouse model, providing proof-of-concept for addressing the UPR as therapeutic target for the treatment of primary tauopathies [50].

In this study we detected profound levels of the UPR activation marker pPERK, the GVD marker CK1δ and neurofibrillary tau pathology in the frontal cortex of a patient presenting an unusual GSS phenotype (case #14). Genetic analysis of this patient revealed a premature stop codon mutation (Q227X) that resulted in a truncated prion protein devoid of the GPI anchor that normally facilitates implementation of the prion protein in the cell membrane [39]. Previous studies in cellular and murine models of genetic prion disease have shown delayed maturation, prolonged retention and accumulation of mutant PrP in the ER, which could be a primary cause of ER stress [11, 15]. The absence of Aβ deposits in the frontal cortex of case #14 indicates that the severe tau pathology is related to the unusual PrP pathology rather than to concomitant AD pathology. Neurofibrillary tau pathology has been documented earlier in other rare hereditary prion disease cases in which mutations in *PRNP* cause truncation of PrP and absence of the GPI anchor [51, 52] as well as other forms of GSS caused by distinct *PRNP* mutations [53–55]. Notably, the presence of GVD was previously mentioned by a case report on a longstanding GSS patient (P102L) with pathological features comparable to case #14, showing neurofibrillary tau pathology secondarily induced by prion amyloidosis [53]. Molecular properties of the prion protein itself as well as the duration of the clinical course could be requisites for the emergence of neurofibrillary tau pathology and UPR activation in human prion diseases.

Data obtained from some experimental models of prion diseases suggest an involvement of the UPR in prion disease pathology. The expression levels of BiP and several other ER chaperones are (transiently) increased in N2A neuroblastoma cells after treatment with PrP^Sc^ derived from scrapie-infected mice and in several brain regions in a murine scrapie model [12, 14]. In addition, the UPR was implied as part of the pathological process of prion disease in a study detecting upregulation of BiP in RNA samples derived from the brainstem of BSE-infected cattle [16]. In contrast, another study did not detect increases in BiP and CHOP expression and XBP1-splicing, a downstream effect of IRE1α activation, in the brains of mice transgenic for mutant PrP and in primary neurons and HEK293 cells transfected with mutant PrP conformers [56]. Discrepancy between pathogenic mechanisms in play in experimental models of prion disease may arise from experimental methodology, e.g. by differences in strain and titer of the administered prions and the use of PrP^C^ overexpressing models.

One of the down-stream effects of UPR activation is a reduction of protein synthesis through phosphorylation of the eukaryotic initiation factor 2α (eIF2α), as a results of increased kinase activity of PERK. Prion infection of PrP overexpressing transgenic mice increases the levels of UPR activation markers, including pPERK and phosphorylated eIF2α [18]. In these mice, reduction of eIF2α phosphorylation rescues synaptic deficits, neuronal loss, and increases survival of prion-diseased mice [18]. In addition, oral administration of a kinase inhibitor of PERK or the small molecule ISRIB, which restores translation downstream of eIF2α, conferred neuroprotection in these prion-diseased mice [57, 58]. The discrepancy between on the one hand indications that the PERK pathway is activated in prion-diseased mice and on the other hand the absence of markers indicative of PERK activation in human prion disease (this study and [28]) could be attributed either to species differences or to the experimental methodology used for prion-diseased mice. Nevertheless, the involvement of the UPR in prion disease models and the therapeutic potential of targeting the UPR observed in these models contribute to understanding the role of the UPR in protein misfolding diseases.

A possible caveat of this study could be an effect of the post-mortem delay on the preservation of phosphorylated antigens that are needed in order to detect UPR activation by immunohistochemistry. However, this concern is undermined by i) the clear presence of pIRE1α and pPERK in the single prion disease cases and AD positive controls that do show immunoreactivity with comparable handling and post-mortem delay, ii) by the marked detection of pIRE1α and pPERK immunoreactivity in AD, but not human prion disease, hippocampus of cases with a post-mortem delay comparable to about half of the human prion disease cases included in this study, iii) by the detection of phosphorylated tau using the phospho-specific AT8 antibody in almost all prion disease cases and iv) by the use of CK1δ as non-phospho-specific surrogate marker of UPR activation.

## Conclusion

In conclusion, our data suggest that the IRE1α branch of the UPR is not implied in human prion diseases. In addition, we confirm earlier data on the absence of increased levels of pPERK in human prion disease in an extensive independent cohort [28]. Furthermore, in this study we report no profound increase in CK1δ immunoreactive structures that resemble GVD in human prion diseases. Since UPR and other markers for cellular stress are detected alongside in neurons undergoing GVD (for review see [27]) future research should address the functional relation between these markers in order to understand the pathogenesis of GVD. In regards to prion disease, future research should focus on the involvement of alternative UPR activation markers in post-mortem brain tissue and atypical activation of the UPR in prion disease models. Results obtained in prion-infected mice indicate alongside the involvement of the PERK pathway in neurodegeneration, that pharmacological inhibition of this pathway is a potential therapeutic target [57, 58]. While available data on UPR activation in human prion diseases remain limited, this type of neuropathological data is essential for proper interpretation of recent therapeutic advances in the field of prion disease.

## Additional files

Additional file 1: Figure S1. Semi-quantitative analysis of the Aβ (IC16) and phosphorylated tau (AT8) burden. Representative examples from our cohort showing the amount of Aβ deposits, small phosphorylated tau positive extracellular inclusions and neuritic changes related to prion disease and phosphorylated tau positive tangle-like structures, most often resulting from AD pathology, corresponding to the semi-quantitative scores +, ++ and +++. Aβ images were taken with the 5× objective, tau (positive inclusions/neuritic changes) images with the 10× objective, tau (tangle-like changes) images with the 20× objective. In Table 3 scores for each case are listed. Scores with a/, e.g. +/++, have a burden in-between the classes represented here. Brown staining with DAB, blue staining of the nucleus with haematoxylin. (PDF 1437 kb)
Additional file 2: Figure S2. Immunohistochemical detection of PrP, pIRE1α, pPERK, and CK1δ in brain tissue of various human prion disease subtypes. Representative pictures of the immunohistochemical detection of PrP (3F4 antibody), pIRE1α, pPERK and CK1δ in frontal cortex sections of human prion disease patients with different disease subtypes, namely GSS (case #10), VPSPr (case #56), sCJD (case #27), panencephalopatic CJD (case #55), PrP-CAA (case #15), FFI (case #18), vCJD (case #21) and iCJD (#24), showing the absence of these UPR activation and GVD markers in the presence of PrP deposition. Immunohistochemical detection is visualized by DAB (brown staining) and nuclei are counterstained with haematoxylin (blue staining). *Bar* 200 μm. (PDF 2131 kb)
Additional file 3: Figure S3. Immunohistochemical detection of pIRE1α and pPERK in AD and CJD hippocampus. Immunohistochemical detection for pIRE1α and pPERK was performed on an AD (case #57) and CJD (case #42) brain tissue with identical post-mortem handling and delay. Pictures from the subiculum are shown. Immunohistochemical detection is visualized by DAB (brown staining) and nuclei are counterstained with haematoxylin (blue staining). *Bar* 50 μm. (TIF 21347 kb)
