# Supplementary figures and images for: Activation of the unfolded protein response and granulovacuolar degeneration are not common features of human prion pathology
Source: Acta Neuropathol Commun. 2016 Oct 28;4:113. doi: 10.1186/s40478-016-0383-7 (PMC5086055; doi:10.1186/s40478-016-0383-7)

Additional file 1: Figure S1

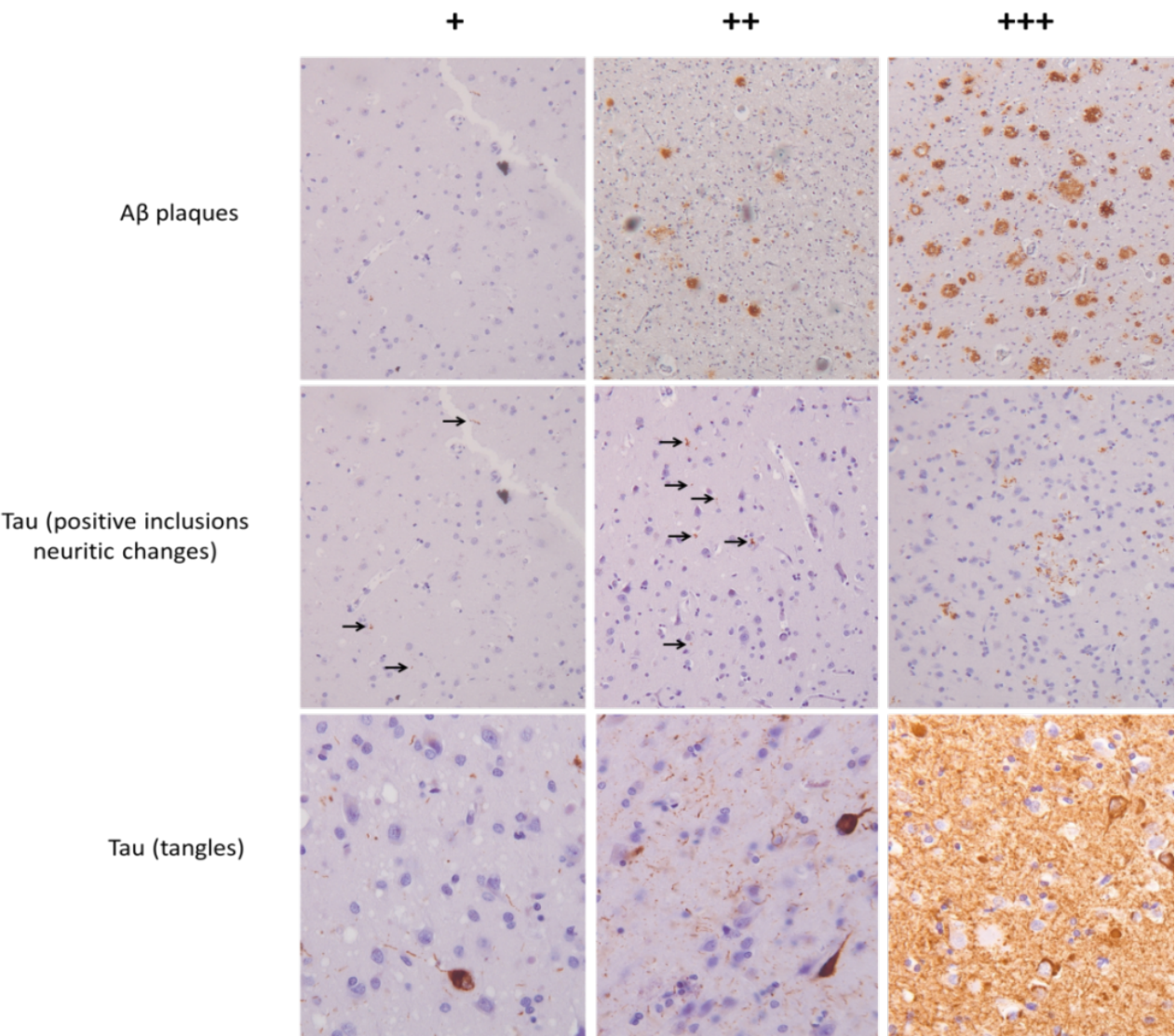

Supplement: Additional file 1: Figure S1. — Semi-quantitative analysis of the Aβ (IC16) and phosphorylated tau (AT8) burden. Representative examples from our cohort showing the amount of Aβ deposits, small phosphorylated tau positive extracellular inclusions and neuritic changes related to prion disease and phosphorylated tau positive tangle-like structures, most often resulting from AD pathology, corresponding to the semi-quantitative scores +, ++ and +++. Aβ images were taken with the 5× objective, tau (positive inclusions/neuritic changes) images with the 10× objective, tau (tangle-like changes) images with the 20× objective. In Table 3 scores for each case are listed. Scores with a/, e.g. +/++, have a burden in-between the classes represented here. Brown staining with DAB, blue staining of the nucleus with haematoxylin. (PDF 1437 kb) [file 40478_2016_383_MOESM1_ESM.pdf]

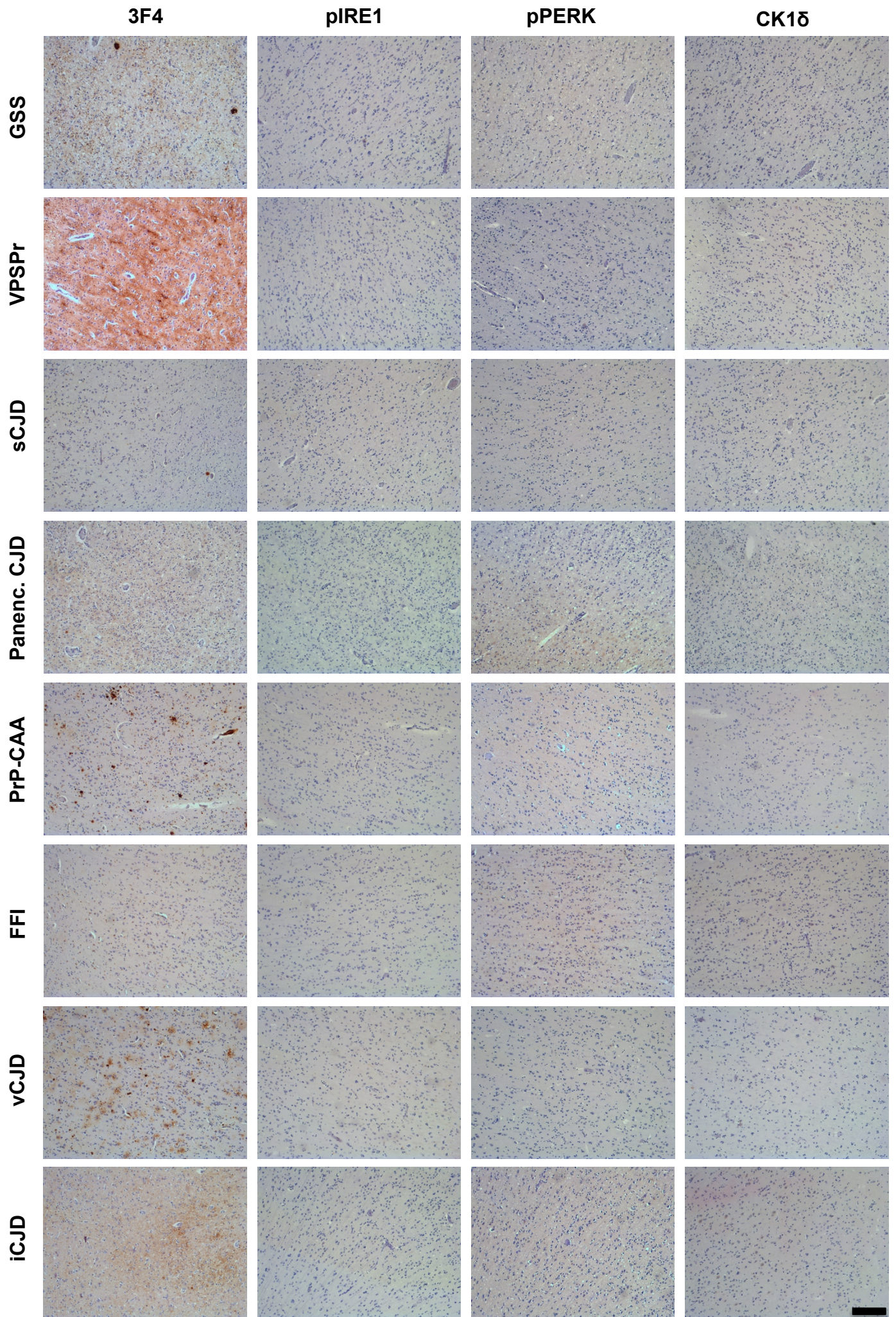

Supplement: Additional file 2: Figure S2. — Immunohistochemical detection of PrP, pIRE1α, pPERK, and CK1δ in brain tissue of various human prion disease subtypes. Representative pictures of the immunohistochemical detection of PrP (3F4 antibody), pIRE1α, pPERK and CK1δ in frontal cortex sections of human prion disease patients with different disease subtypes, namely GSS (case #10), VPSPr (case #56), sCJD (case #27), panencephalopatic CJD (case #55), PrP-CAA (case #15), FFI (case #18), vCJD (case #21) and iCJD (#24), showing the absence of these UPR activation and GVD markers in the presence of PrP deposition. Immunohistochemical detection is visualized by DAB (brown staining) and nuclei are counterstained with haematoxylin (blue staining). Bar 200 μm. (PDF 2131 kb) [file 40478_2016_383_MOESM2_ESM.pdf]

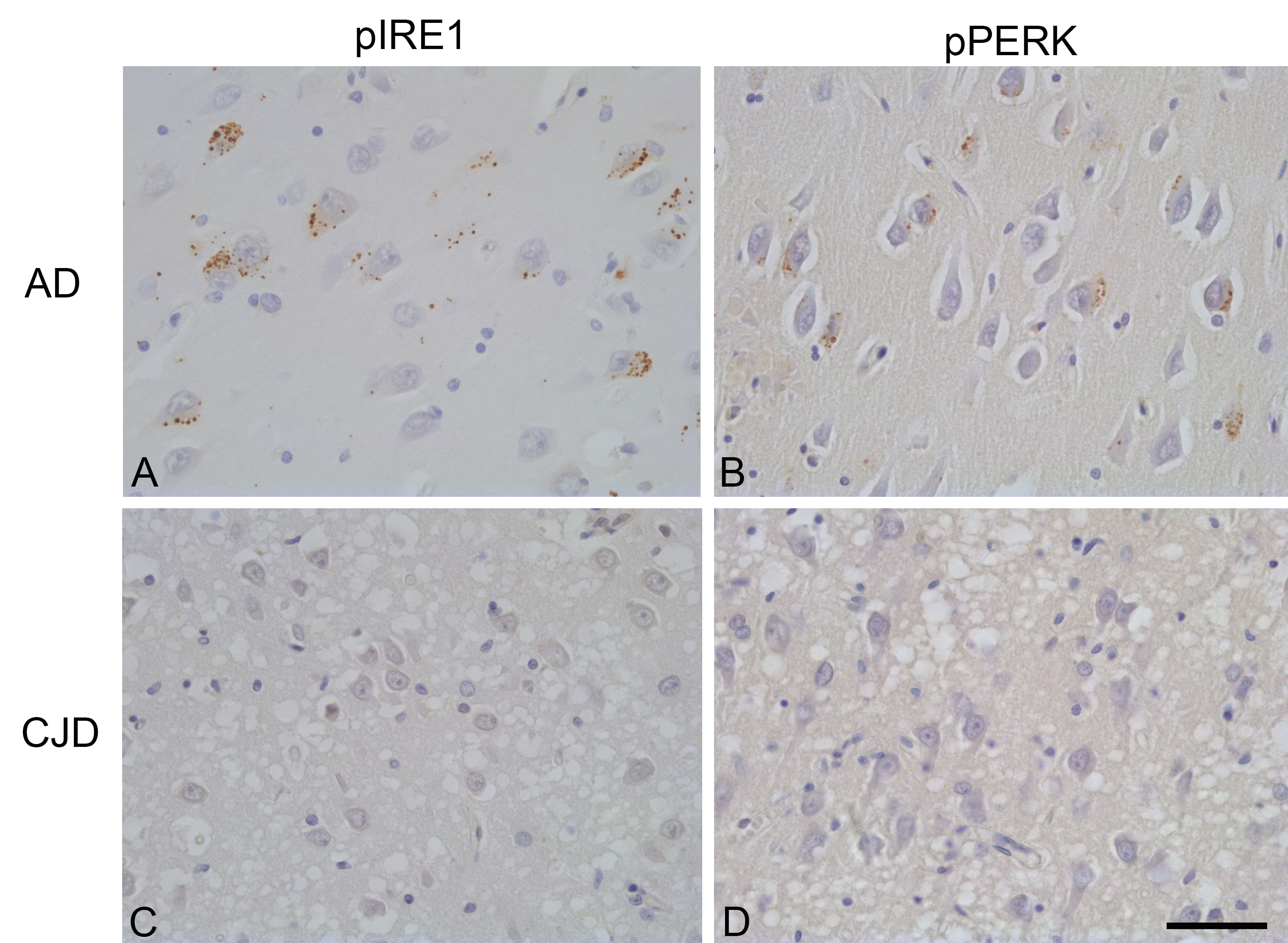

Supplement: Additional file 3: Figure S3. — Immunohistochemical detection of pIRE1α and pPERK in AD and CJD hippocampus. Immunohistochemical detection for pIRE1α and pPERK was performed on an AD (case #57) and CJD (case #42) brain tissue with identical post-mortem handling and delay. Pictures from the subiculum are shown. Immunohistochemical detection is visualized by DAB (brown staining) and nuclei are counterstained with haematoxylin (blue staining). Bar 50 μm. (TIF 21347 kb) [file 40478_2016_383_MOESM3_ESM.tif]
